# Enhanced Osteogenic Differentiation of Primary Human Osteoporotic Osteoblasts on a Roughened Titanium Surface by Vitamin K2 and Vitamin D3 Compared to the Differentiation Behaviour of Primary Healthy Human Osteoblasts

**DOI:** 10.3390/jfb17060288

**Published:** 2026-06-09

**Authors:** Katharina Tscheu, Katharina Schultz, Christoph V. Suschek, Uwe Maus

**Affiliations:** 1 Clinic for Orthopaedics and Trauma Surgery, University Hospital of Düsseldorf, 40225 Düsseldorf, Germany; 2 Clinic for Orthopaedics, Medical University Lausitz—Carl Thiem, 03048 Cottbus, Germany

**Keywords:** osseointegration, primary human osteoblasts, endoprosthesis, osteoporosis, rough surface structure, vitamin K2, vitamin D3, adherence behaviour, osteogenic differentiation

## Abstract

The number of patients who require endoprosthetic treatment related to osteoporosis has increased in recent years. Vitamin D3 supplementation has long been standard practice in osteoporosis treatment, while vitamin K2 has gained importance. Using our in vitro model, we compared the osteogenic behaviour of primary healthy human osteoblasts (hOBs) and primary osteoporotic human osteoblasts (hopOBs) under unchanged conditions, with vitamin K2, vitamin D3 and the combined addition. Fluorescence microscopy examinations on a plastic surface and a rough titanium surface structure revealed morphological differences. A quantitative analysis of mineralisation and differentiation was performed using an alizarin red S assay and analysis of alkaline phosphatase activity. It was shown that the hopOBs behave differently morphologically on the titanium surface, while hopOBs are particularly noticeable due to the higher number of cell–cell interactions with vitamin K2. The rough surface led to more pronounced mineralisation of the hopOBs. This effect was pronounced under vitamin K2. Vitamin D3 had an effect in the initial phase of hopOB differentiation. Overall, vitamin K2 had a greater influence on the mineralisation of hopOBs than expected. It must be assumed that vitamin K2 plays a significantly greater role in the metabolism of hopOBs than previously assumed.

## 1. Introduction

The number of people with osteoporosis has grown rapidly in recent years as a result of demographic change. The reasons for the development of osteoporosis are varied and mostly multifactorial [1]. The disease itself can also be described as a ‘silent’ disease, as osteoporosis does not cause any symptoms such as pain. Patients therefore do not have the classic subjective feeling of illness. As a metabolic disease, osteoporosis only becomes apparent when an event such as inadequate trauma occurs, which nevertheless leads to a fracture [2]. Postmenopausal women in particular have a significantly increased risk of suffering a fracture [3]. The disease can be regarded as an imbalance in bone metabolism, whereby the bone-degrading processes gain the upper hand. Bone mass is thus lost [4]. Appropriate screening and prevention are therefore essential to prevent consequences. In a clinical context, osteoporosis is defined as reduced bone density, which is measured using Dual-Energy X-ray Absorptiometry (DXA). A bone density of 2.5 standard deviations or more below the age reference group is defined as ‘osteoporosis’. Values between 1 and 2.5 standard deviations below are defined as ‘osteopenia’ [5,6].

If a fracture occurs, typically a femoral neck fracture, it is often necessary to treat it with an endoprosthesis [7]. Bone quality and bone density are also crucial for the sufficient anchoring of an endoprosthesis [8]. Other factors that influence the osseointegration of the endoprosthesis include the material and properties of the prosthesis itself, but also the ability of the bone to adapt to new circumstances. One of the most common reasons for surgical revision of implanted endoprostheses is mechanical loosening [9]. In particular, prosthesis movement at an early stage after implantation represents a major risk factor for subsequent revision surgery [10,11,12]. Sufficient anchoring of the prosthesis in the bone is therefore crucial from the outset. In addition, low bone density poses an increased risk of periprosthetic fracture, which necessitates further surgical intervention [13].

The term ‘osseointegration’ now refers to the process in which bone cells bond with implants that have been inserted into the bone. An implant can be described as osseointegrated when there is no longer any relative movement between the implant and the bone cells [14]. The bone’s ability to remodel is crucial to the osseointegration process. This describes the bone’s ability to coordinate the activity of osteoclasts and osteoblasts [15]. If both processes, bone resorption and bone formation, are in balance, a constant bone mass is maintained. In healthy bones, both processes occur regularly to replace old bone with newly formed bone cells and repair minor damage to the bone. In osteoporotic bone, the activity of osteoclasts is increased, leading to a reduction in bone substance [16]. To prevent progressive loss of bone mass, these processes must be influenced. However, the regenerative capacity of osteoporotic bone is reduced but the properties of the prosthesis can be influenced.

Vitamin D has been used for many years to prevent osteoporosis. The active form of the vitamin, vitamin D3, significantly supports the formation of new osteoblasts and activates bone mineralisation [17]. This occurs via several mechanisms, both indirect and direct. On the one hand, vitamin D3 can directly activate osteoblasts [18]. On the other hand, bone metabolism is also regulated by influencing calcium and phosphate balance [19]. Numerous studies have shown that vitamin D can delay and even prevent the development of osteoporosis in the long term [20]. Vitamin D deficiency, on the other hand, can significantly contribute to the development of osteoporosis. The development of osteoporosis is therefore closely associated with vitamin D levels. In healthy bones, additional vitamin D3 supplementation leads to the formation of more extracellular matrix (ECM) and promotes the osteogenic behaviour of osteoblasts [21].

Furthermore, an association between low vitamin K2 blood plasma levels and an increased number of fracture events was also demonstrated [22]. At the same time, oral supplementation with vitamin K2 appears to reduce the likelihood of fractures occurring [23]. The mechanism behind this appears to be that the active metabolite of vitamin K influences the activity of osteoclasts depending on its dosage. The higher the dose, the more likely the differentiation markers of osteoclasts are inhibited [24]. In addition, vitamin K2 has a positive effect on osteocalcin, a fundamental component in the formation of ECM, and thus has a direct influence on the differentiation process [25,26]. However, the exact influence of vitamin K2 on the proliferation and differentiation processes of human bone cells is not yet fully understood [27].

In the treatment and prevention of osteoporosis, the aim is now to combine the oral intake of vitamin D3 and vitamin K2, as vitamin K2 further supports the effects of vitamin D3 [28].

For the first time, this study takes a detailed look at the behaviour of primary human osteoporotic cells in direct interaction with the various components consisting of rough surface structure and both vitamins. Based on our previous work [29] with primary healthy human osteoblasts as a reference, a direct comparison can therefore be made to identify the points at which the two tissue types differ from each other.

## 2. Materials and Methods

To ensure comparability, the same materials and methods were used as those described in our previous paper [29].

### 2.1. Titanium Preparations

Titanium preparations (provided by Peter Brehm GmbH, Weisendorf, Germany) were also used for these in vitro investigations, which exhibited the same characteristics as in our previous investigations [29]. For these investigations, only the rough side of the titanium plates, which were made of the TiAl6V4 alloy, was used. In a clinical context, the rough side is the structure that comes into contact with the bone cells and is therefore clinically relevant. As already shown by scanning electron microscopy [29], the rough side presented an irregular structure with an Rz value of 40–60 μm. Furthermore, the titanium plates were used multiple times and prepared after each test using a by us successful established cleaning protocol [29]. To this end, the specimens were rinsed with acetic acid after each test and left to soak in it for 60 min. They were then left to soak in distilled water overnight. After this chemical cleaning process, they were cleaned mechanically in an ultrasonic bath for 15 min before being autoclaved at 128 °C.

### 2.2. Characteristics of the Cells and the Process of Cell Harvesting

Both primary healthy human osteoblasts (hOBs) and primary osteoporotic osteoblasts (hopOBs) were taken from femoral heads that had to be removed from patients due to, for example, existing osteoarthritis or a fracture. All tissue samples were taken at the University Hospital Düsseldorf in the Department of Orthopaedics. The procedure described was duly authorised by the Research Ethics Committee of the Heinrich Heine University Düsseldorf (Study No. 5585R). In addition, all patients were informed about the procedure and the use of the harvested cells and gave their consent. Prior to harvesting the femoral heads, all patients underwent bone density testing, which was performed on both the lumbar spine and the femoral neck. There was a medical indication for each of these measurements. The measurement was performed using DXA. Patients with a measured T-score of <−2.5 were classified as ‘osteoporotic’. Patients with healthy bones had T-scores of >−2.5 [5], like it is defined in general [6]. The previously determined DXA values of patients whose bone cells were classified as osteoporotic and used as hopOBs ranged from −2.7 to −2.9. Cells from both male and female patients were used. While the age range of the hOBs used was 24 to 86 years, the age distribution of the hopOBs used was narrower due to the classic age of onset of osteoporosis. The cells used were from patients between the ages of 58 and 76.

The cell harvest was carried out using the same procedure for both cell types. After removal of the femoral heads in the operating theatre, they were transported immediately to the laboratory or stored at 4 °C for a certain period of time before being used. For the actual cell harvesting from the femoral heads, the first step was to separate the spongiosa from the compacta using a sharp spoon. The individual parts obtained were transferred to a tube into which 0.25% collagenase type IV (Life Technologies Ltd., Thermo Fisher Scientific, Waltham, MA, USA) in sterile washing medium (1% Penicillin/streptomycin 10,000 U/mL/10 mg/mL in GibcoTM Ham’s F-12 Nutrient mix from Life Technologies Ltd., Thermo Fisher Scientific, Waltham, MA, USA) was filtered. The suspension obtained in this way was incubated for 2 1⁄2 h at 37 °C with continuous shaking. The solution was afterwards transferred to a new tube and centrifuged for 5 min at 400× *g*. This allowed two phases to be distinguished from each other. The supernatant was pipetted off and the pellet was resuspended with the described washing medium and centrifuged a second time under the same conditions. The supernatant was again carefully removed and the cell pellet was resuspended with standard cell medium (DMEM, (Life Technologies Ltd., Thermo Fisher Scientific, Waltham, MA, USA) and finally transferred to small T75 cell bottles [30,31].

#### Cultivating and Differentiation During the Experiments

Both cell types were stored in cell bottles of different sizes in preparation for the experiments. Incubation took place in a classic standard cell culture medium, which was additionally supplemented with 10% foetal bovine serum (Sigma-Aldrich Co., St. Louis, MO, USA), 5% Hepes (Sigma-Aldrich Co., St. Louis, MO, USA) and 5% penicillin/streptomycin (PAN Biotech, Aidenbach, Germany). The incubation conditions were kept constant in the incubator at 37 °C, 5% CO_2_, 100% humidity and in complete darkness. In order to create the best possible growth conditions, all cells in cell culture bottles were regularly checked using a light microscope. The growth progress and vitality of the cells were continuously documented. Every second day, the cell medium was also changed and replaced with fresh medium to ensure the best possible nutrient supply and prevent possible contamination. As soon as it became apparent under the light microscope that the cell culture bottles were covered by an almost continuous layer of cells, and it could therefore be assumed that newly forming cells would have no space and no contact with the surface of the cell culture bottle, the cells were divided among several cell culture bottles. The division of the cells followed a standardised protocol, in which the remaining cell culture medium was first carefully removed. The cell culture bottle was then cleaned with Dulbecco’s Phosphate-Buffered Saline, modified, without calcium, chloride and magnesium (PBS; Sigma-Aldrich Co., St. Louis, MO, USA) to ensure that the cell culture medium had been completely removed. This was followed by the mobilisation of the cells using a mixture of PBS and 10% trypsin (Sigma-Aldrich Co., St. Louis, MO, USA). The cells were incubated with this solution for 5 min under the standard incubation conditions described above. A light microscope was then used to check whether all cells had detached from the cell culture flask. If this was not the case, a soft cell scraper was used to mobilise the remaining cells. The same amount of cell culture medium as previously used for the trypsin solution was added to prevent further mobilisation of the cells. The entire liquid containing the dissolved cells was transferred to a tube and centrifuged at 300× *g* for 5 min. This allowed the liquid to be separated from the solid cells and the resulting cell pellet to be resuspended in cell culture medium. For precise distribution of the cells, a cell count was performed using a Neubauer counting chamber and the cells were evenly redistributed among several cell culture bottles.

All experiments were performed in 24-well plates. In each experiment, cells in native plastic wells were also included as a control group for comparison with the various groups on the titanium preparations. In addition, two plates without any cells were always included to serve as blanks and for comparison. All cells were transferred from the cell culture bottles to the wells containing the plates using the same principle as described above. Initially, 20,000 cells were seeded in 1 mL of standard cell culture medium in each well. The liquid volume of 1 mL was necessary to ensure that the plates were completely surrounded by liquid and that the cells on the plates were also covered with cell culture medium. After 72 h of growth in the standard cell culture medium, this was carefully removed and replaced with differentiation medium. In contrast to the standard cell culture medium, this was not supplemented with 5% Hepes, but with dexamethasone, L-ascorbic acid 2-phosphate and ß-glycerophosphate (all from Sigma-Aldrich Co., St. Louis, MO, USA) in addition to foetal bovein serum and penicillin/streptomycin [32]. Depending on the experiment, the differentiation medium was also supplemented with vitamin K2, vitamin D3 (both from Sigma-Aldrich Co., St. Louis, MO, USA) or both vitamins. To create an optimal environment for cell differentiation, the differentiation medium (with or without the vitamins) in each well was carefully changed every 48 h.

### 2.3. Quantitative Analysis of the Differentiation Capacity and Development of the Extracellular Matrix (ECM)

Two different methods were used to quantitatively measure differentiation capacity.

#### 2.3.1. Alkaline Phosphatase (ALP)

The analysis of the enzymatic activity of ALP served as an osteogenic marker to demonstrate the differentiation capacity. The evaluation was carried out according to a standardised protocol. At the predetermined evaluation time, the medium still present on the cells or platelets was first carefully removed and then any remaining medium was also removed by adding PBS. After the PBS had also been removed, the cells were incubated with 4-nitrophenol solution (Sigma-Aldrich Co., St. Louis, MO, USA) for 20 min at room temperature and in complete darkness. Samples were taken from each well and transferred in duplicate to a 96-well plate. The actual measurement of the values was performed at 405 nm in a microplate reader. For evaluation, the average value of the titanium preparations without cells was subtracted from the values obtained for the titanium preparations with cells [33].

#### 2.3.2. Alizarin Red S Staining

To quantify the degree of ECM expression, analysis is performed using Alizarin Red S staining. This works because the calcium stored in the ECM forms complexes with alizarin. The more calcium is stored, the more complexes can be formed and the higher the final colour intensity measured [34]. To perform the assay, the cell culture medium was first removed at the time of evaluation and the wells were washed with PBS to completely remove the platelets and cells from the cell culture medium. The cells on the platelets and in the native well were then fixed with ROTI^®^ Histofix (Carl Roth GmbH + Co.KG, Karlsruhe, Germany) and incubated in the dark for 15 min at 37 °C. The excess fixing fluid was removed with distilled water so that the actual staining could be carried out with 0.5% Alizarin Red Smonosodium salt in distilled water for 20 min at 37 °C. The cells were washed three times with distilled water before the staining solution was removed. To do this, 10% cetylpyridinium chloride solution (Sigma-Aldrich Co., St. Louis, MO, USA) was added and the well plates were shaken on the mini plate shaker (type KM2, Edmund Bühler GmbH, Bodelshausen, Germany) for 60 min to ensure that the staining solution was completely removed, even from the rough side of the plates, without having to mechanically treat the surface of the plates. Finally, the colour intensity of the solution was measured in the microplate reader at 450 nm. For evaluation, the mean value of the native platelets was subtracted from the values obtained for the platelets with cells.

### 2.4. Immunofluorescent Visualisation of Cell Development with Calcein Staining

After 1 and 7 days of differentiation, immunofluorescent images were taken using calcein staining to qualitatively represent the expression of the ECM. Calcein is mainly used to represent the degree of calcification and the expression of calcium deposits in the ECM, as the calcein added in the staining binds with the deposited calcium carbonate [35,36]. For better representation and visualisation of cell distribution, and to better distinguish individual cells, cell nuclei staining with Hoechst was also performed.

The established analysis was performed according to a developed protocol, in which the cell culture medium was first removed from the cells on the plates and then carefully washed with PBS. The actual staining was performed using a calcein solution consisting of calcein (ApolloScientific Ltd., Stockport, UK) and PBS, whereby the cells were incubated with this solution for 20 min in the dark at room temperature. To remove the excess solution, the cells were then washed three times with PBS and Hoechst (ThermoFisher Scientific Ltd., Waltham, MA, USA) was added to stain the cell nuclei. This was followed by further incubation for 5 min at room temperature and in the dark before the cells could be visualised under a fluorescence microscope (Axiovert 200; Carl Zeiss AG, Jena, Germany).

### 2.5. Statistical Analysis

The software Graph Pad Prism (version 10.3; Boston, MA, USA) was used for statistical analysis. In the analyses, the level α = 0.05 was used to remove the first-order error. The selected significance level was 5% (*p* < 0.05). To present the results in a way that allows for better comparability within a cell line, box plots were selected, showing the average value of each group and the standard deviations. The comparison between the cell lines, on the other hand, was carried out using line graphs. The significance level of 5% is indicated by one asterisk. Two asterisks indicate *p* < 0.01 and three asterisks indicate a level of *p* < 0.001. Whether these differences are significant was determined using the *t*-test and the Mann–Whitney U-test.

## 3. Results

### 3.1. Differentiation and Mineralisation

#### 3.1.1. ALP (Figure 1)

In the early phase of hopOB differentiation, significantly more pronounced differentiation was observed on the rough side of the titanium preparations when treated with vitamin D3 alone compared to the addition of vitamin K2 alone. The combined addition of both vitamins also resulted in significantly increased differentiation compared to differentiation with the addition of vitamin K2 alone. The combined vitamin addition also resulted in significantly higher differentiation than on the native titanium preparations. After 7 days of differentiation, the degree of differentiation was already more pronounced on the native titanium preparations than on the native plastic well. The addition of vitamin K2 alone and the combined addition of both vitamins resulted in significantly more pronounced differentiation compared to the differentiation on the native preparations. In contrast, differentiation without the addition of vitamins in combination with the titanium preparations was significantly more pronounced than under treatment with vitamin D3. The combined addition of vitamin K2 and vitamin D3 showed a significantly higher degree of differentiation than the addition of vitamin D3 alone than the addition of vitamin K2 alone.

**Figure 1:**
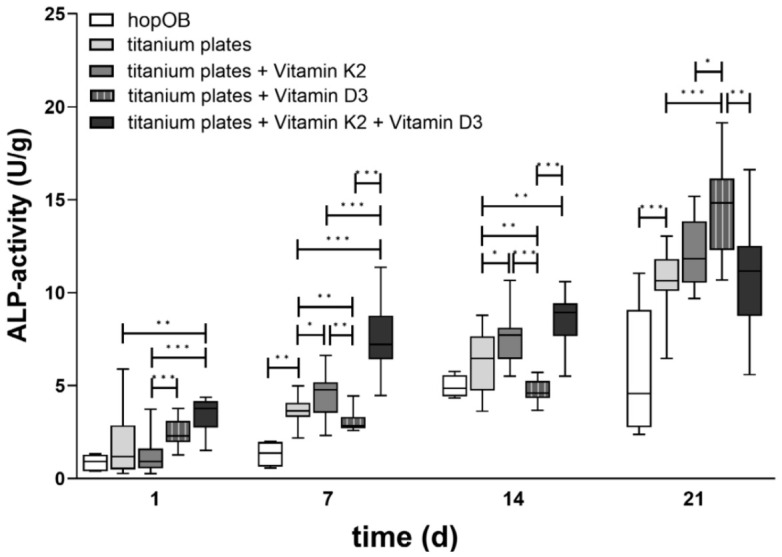
The figure shows the change in ALP activity in differentiated hopOBs. For comparison, it illustrates the trends in ALP activity in native plastic culture medium (white), on the rough titanium surface without the addition of vitamins (light grey), on the rough titanium surface with the addition of vitamin K2 (grey), on the rough surface with the addition of vitamin D3 (black with white stripes) and on the rough surface with the combined addition of both vitamins (black). The significance levels are marked with stars, whereby * *p* < 0.05, ** *p* < 0.01, *** *p* < 0.001.

After 14 days of differentiation, vitamin K2 treatment resulted in significantly increased ALP activity compared to the ALP activity of hopOBs on native titanium preparations and compared to ALP activity under vitamin D3. In contrast, significantly more pronounced differentiation was observed on the native titanium preparations compared to the degree of differentiation of the hopOBs under vitamin D3 addition. The combined addition of both vitamins resulted in significantly higher differentiation compared to the degree of differentiation on the native titanium preparations and the differentiation under vitamin D3.

Long-term observation already showed a significant difference in differentiation between the hopOBs in the native well and the differentiated hopOBs on the native titanium preparations in favour of differentiation on the titanium surface. The addition of vitamin D3 alone resulted in significantly higher ALP activity compared to the native titanium preparations, the addition of vitamin K2 alone, and the combined addition of both vitamins.

#### 3.1.2. Comparison of the ALP Activity Between hopOBs and hOBs

**Figure 2:**
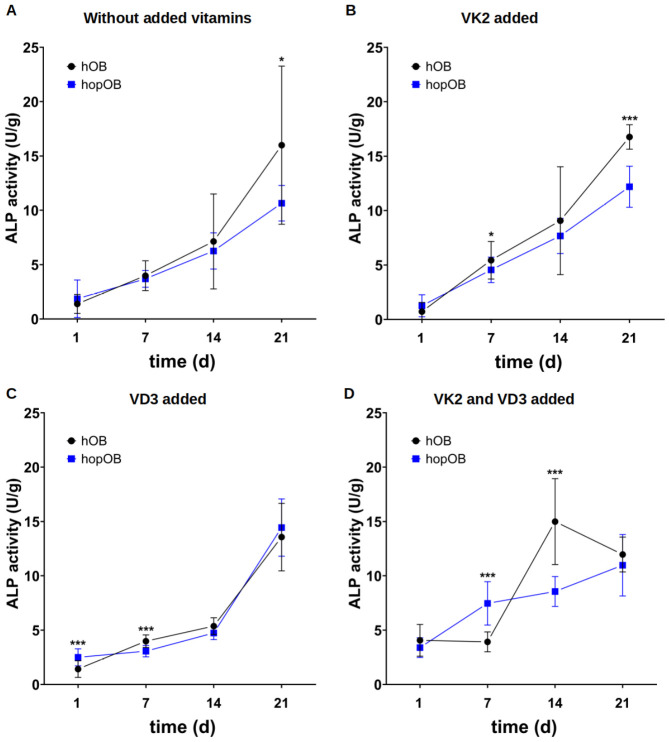
Comparison of ALP activity between differentiated hOBs (black circles) and differentiated hopOBs (blue squares) over the time on the rough titanium surface under native conditions (**A**), with the addition of vitamin K2 (**B**), vitamin D3 (**C**) and the combined addition of both vitamins (**D**). The asterisks indicate the different significance levels. * *p* < 0.05, *** *p* < 0.001.

When comparing ALP activity between hopOBs and hOBs, it was noticeable that on day 21, even without the addition of vitamins, there was a significantly higher differentiation of hOBs on the untreated rough surface structure. At the previous survey dates, there were no significant differences between the two cell types (Figure 2A). This also applied to the addition of vitamin K2 for long-term observation of differentiation, whereby the difference proved to be highly significant. Furthermore, the addition of vitamin K2 also resulted in significantly more pronounced differentiation of hOBs on day 7 (Figure 2B). However, this was not observed with the addition of vitamin D3. On day 1, in the early phase of differentiation, there was significantly higher differentiation of hopOBs. On day 7, however, the ALP activity of hOBs was significantly increased. No further significant differences were observed in the long-term follow-up (Figure 2C). With the addition of both vitamins, there was initially a significantly more pronounced differentiation of hopOBs compared to hOBs on day 1, while on day 7 the opposite was observed. Under the influence of both vitamins, the maximum ALP activity of hOBs was already evident on day 14 and declined again by day 21, while the maximum differentiation capacity of hopOBs had not yet been reached (Figure 2D).

#### 3.1.3. Alizarin Red S Staining (Figure 3)

After 24 h of differentiation, significantly more pronounced mineralisation of the hopOBs was observed on the rough side of the plates even without the addition of vitamins compared to the hopOBs in the native well. This was also evident after 7 and 21 days of differentiation. Only after 14 days of differentiation was there no significant difference between these two groups. In the initial differentiation phase after 1 day, both under the influence of vitamin D and in the combined addition of both vitamins, there was significantly more pronounced mineralisation on the rough side of the preparations compared to the native mineralisation of the hopOBs on the native titanium platelets. There was also significantly higher mineralisation of the hopOBs under the combined vitamin administration compared to the administration of vitamin K2 alone. This changed after 7 days of mineralisation. Here, mineralisation on the rough side of the titanium preparations was significantly increased under the administration of vitamin K2 alone compared to all other treatments. The same picture was also seen after 14 days. At this point, significantly higher mineralisation was also demonstrated under treatment with vitamin D3 compared to the native titanium preparations. In the long-term observation over 21 days, the mineralisation of the hopOBs was significantly more pronounced under vitamin K2 than under vitamin D3. Similarly, mineralisation was significantly increased under the addition of both vitamins compared to treatment with vitamin D3 alone. No significant difference in the degree of mineralisation between treatment with vitamin K2 alone and combined vitamin supplementation could be established at the time of this survey.

**Figure 3:**
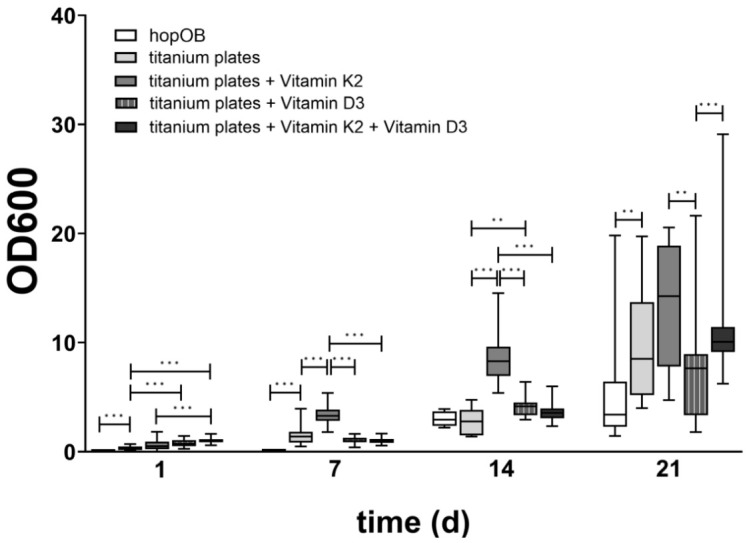
The changes in the degree of mineralisation of differentiated hopOBs over time are shown. White box plots illustrate the degree of mineralisation of hopOBs in the native plastic well. Light grey indicates the degree of mineralisation of hopOBs on the rough titanium surface without the addition of any vitamins, whilst grey shows the degree of mineralisation on the rough surface with the addition of vitamin K2, grey with white stripes represents the degree of mineralisation on the rough titanium surface under the influence of vitamin D3, and black represents the degree of mineralisation on the rough surface with the combined addition of both vitamins. Stars are used to represent the different levels of significance, whereby the significance level of ** *p* < 0.01 and *** *p* < 0.001.

#### 3.1.4. Comparison of the Manifestation of the ECM Between hopOBs and hOB

**Figure 4:**
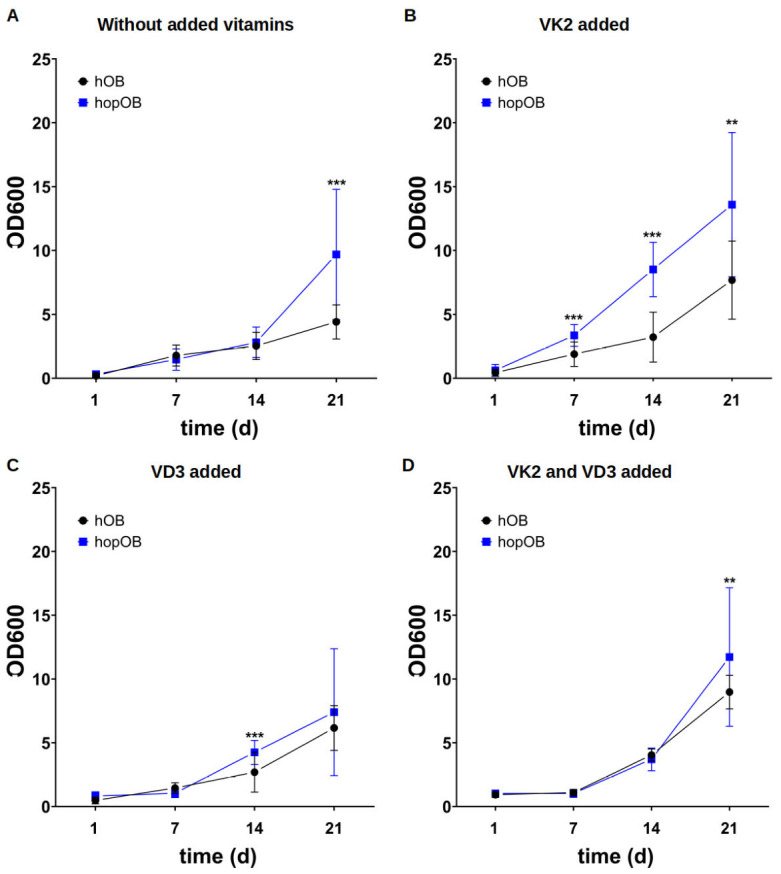
Comparison of mineralisation between differentiated hOBs (black circles) and hopOBs (blue squares) over the time on the rough titanium surface under native conditions (**A**), with the addition of vitamin K2 (**B**), vitamin D3 (**C**) and the combined addition of both vitamins (**D**). The asterisks indicate the different significance levels. ** *p* < 0.01 and *** *p* < 0.001.

The opposite effect due to the described observation of the comparison of the ALP activity was observed in the evaluation of ECM mineralisation using alizarin red S staining. Even without the addition of vitamins, there was significantly more pronounced mineralisation of the ECM of hopOBs on day 21, while no significant differences were observed compared to the previous evaluation days between both cell types (Figure 4A). With the addition of vitamin K2, ECM mineralisation of hopOBs was significantly higher from day 7 onwards. Only in the initial phase of mineralisation on day 1 was there no significant difference in the degree of mineralisation (Figure 4B). When vitamin D3 was added, significantly higher mineralisation was observed in hopOBs compared to hOBs only on day 14. No significant differences were observed at the other evaluation points. (Figure 4C). During treatment with both vitamins, no significant differences in the mineralisation characteristics of both cell lines were observed over the first time of the experiment. At least at day 21, a significant higher mineralisation of the ECM of the hopOBs was shown (Figure 4D).

### 3.2. Visualisation of Cell Adherence and Cell Differentiation

**Figure 5:**
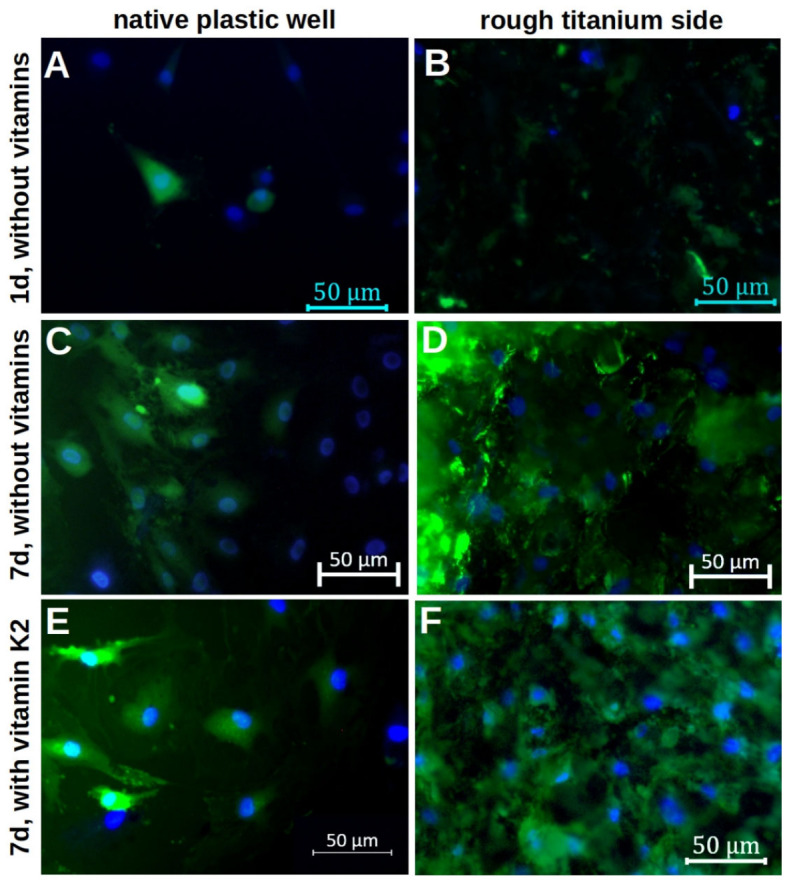
Fluorescence microscopy images of hopOBs cells stained with calcein and Hoechst in native plastic culture medium after 1 day of differentiation without vitamin supplementation (**A**), after 7 days of differentiation without vitamin supplementation (**C**), and after 7 days of differentiation with the addition of vitamin K2 (**E**) compared to hopOBs on the rough titanium surface, in comparison to hopOBs on the rough side of the titanium specimens under the same conditions (**B**,**D**,**F**).

Both hopOBs in the native plastic well and on the rough titanium surface were clearly visible in the fluorescence microscope images at all observation times (Figure 5).

After one day of differentiation without the addition of vitamins, hopOBs cells were sparse and scattered, with a modest amount of surrounding ECM, both in the native plastic well (Figure 5A) and on the rough surface structure of the titanium specimens (Figure 5B). On the rough surface structure, it was notable that ECM was already present in more locations (Figure 5B), whereas in the plastic well this was limited to a few exceptions (Figure 5A).

After seven days of differentiation on both surface structures, the development of ECM was evident in both configurations. In the native well, this was primarily deposited concentrically around the cell nuclei, although this was limited to only a proportion of the cells (Figure 5C). Even at this stage of mineralisation, cell nuclei without surrounding ECM could be clearly distinguished. On the rough surface structure, however, the ECM that had formed appeared more diffuse, and individual cells were difficult to distinguish (Figure 5D).

When vitamin K2 was added to both surfaces, it was observed that ECM formation in the plastic well was enhanced compared to differentiation without the addition of vitamin K2. The ECM extensions appeared significantly more extensive and space-occupying when vitamin K2 was added than without the vitamin supplement (Figure 5E). On the rough surface structure, a dense network of ECM was also evident after 7 days of differentiation with the addition of vitamin K2; however, this appeared less extensive but more stable and interwoven than without the addition of vitamin K2 (Figure 5F). Compared to differentiation in the plastic well (Figure 5E), it was again difficult here to precisely delineate individual cells.

## 4. Discussion

### 4.1. Role of Titanium and the Surface Structure in the Process of Mineralisation and Differentiation of hopOBs

It was demonstrated that the processes of mineralisation (Figure 3) and differentiation (Figure 1) occur more rapidly and to a greater extent on the rough surface structure of titanium than on the native plastic wave. Only at certain points in time did differentiation and mineralisation occur at a similar level in these two groups. It can be assumed from the fluorescent observation (Figure 5) that both the titanium itself and the roughened surface structure have an influence on the differentiation and mineralisation of hopOBs. Even the smallest pores in the surface structure appear to stimulate macrophages in particular, which in turn promote cell differentiation via paracrine signals [37]. The rugged and irregular surface structure can thus be regarded as a driver of differentiation and mineralisation. This confirmed that the surface structure has a significant influence on the osteogenic behaviour of hopOBs. One reason for this may be that the number of cell–titanium contacts is higher on the raised, rough surface structure than on the smooth surface structure, where the cell–titanium contacts are more flat [38]. This general trend was also observed with hOBs [29]. A direct comparison showed that hopOBs mineralised significantly more than hOBs due to the rough titanium surface (Figure 4A). The microenvironment is essential for the successful osseointegration of both cell types and has a direct influence on the development of a stable cell network [39]. Due to a change in the cell morphology of hopOBs [40], these appeared to benefit significantly from the rough surface in terms of their adhesion behaviour.

At the same time, however, it was also observed that the differentiation behaviour of hOBs and hopOBs was very similar in the first observation phase. In the long-term observation, however, it became apparent that the differentiation of hOBs on the rough surface structure was increased compared to hopOBs (Figure 2A). At the same time, even after 21 days of observation, the maximum differentiation had not yet been reached in either cell type. After day 14, however, it could be assumed that the initial differentiation-promoting effect of titanium and the rough surface had decreased somewhat for the hopOBs. One possibility was that the hopOBs initially extracted from the hip head specimens contained a significant number of osteoclasts. Overall, there are indications that titanium itself causes osteoclast activation [41]. In order to examine this aspect more closely, all osteoclasts would have to be removed prior to the investigations, and the same investigations would have to be carried out again with pure osteoporotic osteoblasts. Nevertheless, our findings provided further evidence that there may be an interaction between titanium and osteoclasts.

### 4.2. Osseointegration in Osteoporotic Cellnetworks Influenced by the Vitamins

When comparing the osseointegration achieved between hopOBs and hOBs, it should be noted that the age range of the donors of the cells used is narrower for hopOBs than for hOBs. It is possible that this represents a limitation on the validity of the findings. At the same time, however, it must be taking into account that osteoporosis is a disease of older people and, in women, primarily a condition that develops after menopause [3], meaning that there cannot be any young donors of osteoporotic cells. However, an attempt was nevertheless made to achieve as wide an age range as possible among the hopOBs donors, with the youngest person included being 58 years old.

#### 4.2.1. Vitamin K2

The addition of vitamin K2 had several observable effects. First, vitamin K2 administration resulted in a significantly more pronounced cell network on the rough surface structure (Figure 5F). In general, it was also observed on the smooth surface of the plastic well that, under the influence of vitamin K2, the cell bodies and the surrounding ECM appeared more spread out and with more cell extensions (Figure 5E). This was even more evident on the rough surface structure. Here, the addition of vitamin K2 led to the formation of a dense cellular network that appeared organised (Figure 5F), whereas in the absence of vitamin K2, the ECM that formed appeared flatter and less structured (Figure 5D). At the same time, vitamin K2 significantly increased the mineralisation of hopOBs in the short and long term compared to hOBs (Figure 4B). Only in terms of differentiation were there significant differences in favour of hOBs (Figure 2B). Taken together, it can therefore be concluded that the addition of vitamin K2 alone promotes the formation of the ECM in hopOBs. This effect is further enhanced by the rough titanium surface structure. The combined influence of the rough surface and the addition of vitamin K2 in hopOBs exceeds the effect that the combination of these two factors has on hOBs (Figure 4B). At the same time, this marked effect cannot be observed to the same extent with regard to ALP activity (Figure 2B). It is entirely plausible that ALP activity will continue to rise over observation periods exceeding 21 days and could, in some cases, exceed the ALP activity in hOBs. This warrants further investigation. The precise effects that vitamin K2 appears to have when combined with titanium have not yet been studied in detail. To date, vitamin K2 has been used regularly in Asia for the treatment of osteoporosis, and the optimal therapeutic dosage is still under discussion [42, 43]. This naturally makes it difficult to transfer oral administration to in vitro studies. At the same time, the dosage of vitamin K2 cannot be increased indefinitely, as the vitamin has regulatory effects in numerous parts of the body and can also have toxic effects in the event of an overdose [44].

#### 4.2.2. Vitamin D3

The administration of vitamin D3 was shown to primarily increase the differentiation of hopOBs (Figure 1). Only in the short term did the degree of differentiation of hopOBs exceed that of hOBs, with no significant differences observed in the long term (Figure 2C). At the same time, it was also noted that vitamin D3 had less influence on the mineralisation of hopOBs and only significantly increased the mineralisation of hOBs compared to hopOBs on day 14 (Figure 4C). This shows that the effect of vitamin D3 on the various metabolic processes had a significantly lower influence than assumed.

This observation could be explained by the fact that vitamin D3 influences osteoclastogenesis but not the activity of existing osteoclasts, which means that the effect of vitamin D3 may be delayed [45,46]. Furthermore, it was discussed whether the optimal vitamin D3 dosage was used for the treatment of hopOBs. Oral application studies have shown that a certain amount of vitamin D3 is necessary to achieve a sufficient effect [47]. The optimal intake of the vitamin was also discussed. In a therapeutic context, both bolus doses at weekly intervals and continuous doses are being discussed [48]. In our in vitro studies, bolus doses were simulated at intervals of up to 72 h. It is conceivable that the effect could be increased if a continuous or shorter-interval vitamin supply were used. Application studies have also shown that high-risk patients, especially postmenopausal women, benefit from higher doses of vitamin D3 [49]. However, our studies also included cells from male and younger individuals, who may have benefited less from vitamin D3. Furthermore, the aim should be to determine the optimal dosage for each individual patient, both in oral administration and in vitro research, and to further advance the individualisation of medicine.

#### 4.2.3. Combination of Both Vitamins

Overall, the combination of both vitamins did not show any addition to the effects described above. Although the long-term observation of mineralisation showed a significantly higher incidence of this in hopOBs compared to hOBs (Figure 4D), a clear trend was not observed in the differentiation. Here, hOBs already reached maximum differentiation on day 14, which had not yet been achieved in hopOBs (Figure 2D). Observation beyond day 21 would be appropriate here.

It has been described that vitamin K2 supports the effects of vitamin D3. However, this could not be clearly proven in our studies. Rather, it remains to be discussed to what extent the dosage of vitamins plays a role in influencing osteogenic effects. It is conceivable that both vitamins could be reduced in their respective individual dosages in order to achieve optimal results. At the same time, however, it is also conceivable that a high dosage of vitamin D3 requires only a low dose of vitamin K2, as is the case with oral nutrition, for example [50]. At the same time, however, it seemed to be generally the case that patients benefited most from the combination of both vitamins when taken orally [51]. It should also be noted that calcium and magnesium play an important role in therapeutic applications alongside both vitamins. In this country, calcium is currently regarded as the decisive factor in the treatment of osteoporosis alongside vitamin D3 [52]. As described, a standard cell culture medium with a calcium content of 1.8 mM was used in our in vitro studies [53]. No additional calcium was added. It has been reported that calcium alone is not sufficient to treat osteoporosis [52,54]. At the same time, calcium is considered essential, especially in the third decade of life, to maintain sufficient bone metabolism. Due to the deliberately mixed age structure of the cells we selected from the test subjects, it remains unclear what effect the calcium already present had and what an additional dose of calcium could have [28].

In addition, the concentration and role of magnesium must also be discussed [55,56]. Magnesium serves as a cofactor for numerous enzymatic processes and is essential for normal vitamin D metabolism [57]. In the standard cell culture medium, magnesium was present at a concentration of 0.09767 g/L, but the dosage was not increased further. Here, too, there is a possibility that increasing the dosage could achieve short-term support for the effects of vitamin D3 and vitamin K2.

## 5. Conclusions

The in vitro model we developed was also successfully applied to hopOBs. The cell network formed by hopOBs was densest on a rough titanium surface structure. The mineralisation of hopOBs was increased, particularly in long-term observation, by the titanium material itself and the rough surface texture. Vitamin K2 proved to be the most effective in promoting comprehensive mineralisation in both the short and long term. Compared to hOB, hopOBs benefited even more significantly from vitamin K2. However, the expected dramatic effect of vitamin D3 on mineralisation and differentiation did not materialise. Even when both vitamins were combined, a significant difference was only observed in long-term mineralisation. Further long-term studies with modified dosages and the addition of calcium and magnesium, for example, should therefore be carried out in order to achieve the most pronounced osseointegration possible in osteoporotic tissue in the long term.

## Data Availability

The original contributions presented in this study are included in the article. Further inquiries can be directed to the corresponding author.

## Abbreviations

The following abbreviations are used in this manuscript:

ALP	Alkaline phosphatase
DMEM	Standard cell medium
DXA	Dual-energy X-ray absorptiometry
ECM	Extracellular matrix
hOB	Primary healthy human osteoblasts
hopOB	Primary osteoporotic human osteoblasts
PBS	Phosphate-buffered saline
VD3	Vitamin D3
VK2	Vitamin K2
